# Social Brain Network of Children with Autism Spectrum Disorder: Characterization of Functional Connectivity and Potential Association with Stereotyped Behavior

**DOI:** 10.3390/brainsci13020280

**Published:** 2023-02-07

**Authors:** Yonglu Wang, Lingxi Xu, Hui Fang, Fei Wang, Tianshu Gao, Qingyao Zhu, Gongkai Jiao, Xiaoyan Ke

**Affiliations:** Child Mental Health Research Center, the Affiliated Brain Hospital of Nanjing Medical University, Nanjing 210029, China

**Keywords:** autism spectrum disorder, brain functional network, functional connectivity, social-related brain regions, stereotyped behavior

## Abstract

**Objective:** To identify patterns of social dysfunction in adolescents with autism spectrum disorder (ASD), study the potential linkage between social brain networks and stereotyped behavior, and further explore potential targets of non-invasive nerve stimulation to improve social disorders. **Methods:** Voxel-wise and ROI-wise analysis methods were adopted to explore abnormalities in the functional activity of social-related regions of the brain. Then, we analyzed the relationships between clinical variables and the statistical indicators of social-related brain regions. **Results:** Compared with the typically developing group, the functional connectivity strength of social-related brain regions with the precentral gyrus, postcentral gyrus, supplementary motor area, paracentral lobule, median cingulum, and paracingulum gyri was significantly weakened in the ASD group (all *p* < 0. 01). The functional connectivity was negatively correlated with communication, social interaction, communication + social interaction, and the total score of the ADOS scale (r = −0.38, −0.39, −0.40, and −0.3, respectively; all *p* < 0.01), with social awareness, social cognition, social communication, social motivation, autistic mannerisms, and the total score of the SRS scale (r = −0.32, −0.32, −0.40, −0.30, −0.28, and −0.27, respectively; all *p* < 0.01), and with the total score of SCQ (r = −0.27, *p* < 0.01). In addition, significant intergroup differences in clustering coefficients and betweenness centrality were seen across multiple brain regions in the ASD group. **Conclusions:** The functional connectivity between social-related brain regions and many other brain regions was significantly weakened compared to the typically developing group, and it was negatively correlated with social disorders. Social network dysfunction seems to be related to stereotyped behavior. Therefore, these social-related brain regions may be taken as potential stimulation targets of non-invasive nerve stimulation to improve social dysfunction in children with ASD in the future.

## 1. Introduction

Autism spectrum disorder (ASD) is characterized by impairments in communication, restricted repetitive behaviors, and reciprocal social interaction. The prevalence of ASD has been steadily increasing year by year. Recent epidemiological studies suggest that about 1% of the world’s population suffers from ASD [1,2]. Additionally, according to the Autism and Developmental Disabilities Monitoring (ADDM) data, 1 in 54 children in the United States was affected by ASD in 2020 [3]. Moreover, about 1/3 of the children with ASD are unable to live independently after reaching adulthood, imposing a heavy burden on their families and society. 

The etiology and pathogenesis of ASD are unclear. Studies have suggested that factors such as genetics, family, environment, and biology contribute to the onset of ASD. Studies have found weaker functional integration ability with more involvement of a network of intra-regions over inter-regions in patients with ASD compared to healthy individuals [4,5,6]. Moreover, Harlalka et al. demonstrated higher dynamics of ASD connection strength than in the normal group, implying that ASD patients may need to expend more resources on simple tasks [7]. Meanwhile, many studies have discovered local over-connectivity and cortical under-connectivity in the brains of ASD subjects, which may be one of the causes of ASD [8,9,10,11]. Furthermore, several other studies have also shown that some brain regions—primarily the left hemisphere—are under-activated in ASD patients [12,13]. Recently, a study has also shown that active social interaction improves ASD symptoms [14]. However, the association between social deficits and stereotyped behaviors at the level of brain function is still unclear.

There are still no accurate diagnostic methods or effective treatment approaches for ASD. Functional magnetic resonance imaging (fMRI) is considered to be the best imaging tool for diagnosing ASD. fMRI can detect abnormal brain function in the early stages. Regarding treatment, behavioral analysis therapy has always been the cornerstone of treating ASD-related social dysfunction [15]. The sensory-based hug machine is a deep-pressure device designed to calm hypersensitive persons [16]. However, their use is limited for children with dysfunctional and overactive ASD. 

Transcranial direct-current stimulation (tDCS) is a non-invasive brain stimulation method that has gained increasing importance in recent years as an alternative treatment for neuropsychiatric conditions [17,18]. tDCS is effective in alleviating neuropsychiatric symptoms in patients with ASD [19,20]. In addition, studies have reported that brain function activities contain a lot of ASD-related element information, and fully excavating the potential information can help to formulate better diagnosis and treatment strategies.

Social and communication disorders are the core symptoms in ASD children, which seriously affect the patients’ quality of life and prognosis. Studies have found that the temporoparietal junction area [7,21], posterior inferior temporal sulcus [22], prefrontal cortex [23], anterior cingulate cortex [24], fusiform gyrus (FFG) [25], thalamus (THA), amygdala (AMYG) [26], and hippocampal (HIP) CA2 [27] play an indispensable role in human social interaction. Moreover, some of the above social-related brain regions, such as the prefrontal cortex [20] and temporal parietal junction area [28], are highly involved in social understanding and are the core areas of damage in children with ASD. Furthermore, targeting these regions using transcranial direct-current stimulation (tDCS) may improve social communication among ASD children; its physiological mechanism may be to enhance or weaken the functional connectivity of related brain regions [29]. 

However, according to the current research, the brain regions and brain networks related to social disorders are still not fully understood in children with ASD, which further limits the precise neural regulation of ASD mediated by social brain circuits. Thus, there is an urgent need to explore the social-related brain regions of ASD children and screen suitable targets to achieve the purpose of individualized intervention for social disorders in clinical practice. 

In this study, we used two analytical methods (voxel-wise and ROI-wise analysis) to identify patterns of social dysfunction in adolescents with ASD and further explore the potential linkage between social brain networks and stereotyped behavior. This study can help to deepen the understanding of the abnormalities of the social brain network in ASD, revealing the potential association between social dysfunction and stereotyped behavior, and providing potential targets of non-invasive nerve stimulation to improve social disorders, along with theoretical support for the further exploration of the precise neural regulatory technology mediated by social brain circuits in the future.

## 2. Materials and Methods

### 2.1. Participants

All children were selected from the Specific Disease Cohort of ASD in the Child Mental Health Research Center, the Affiliated Brain Hospital of Nanjing Medical University. The inclusion criteria for the ASD group were as follows: (1) Those who met the ASD diagnostic criteria of the *Diagnostic and Statistical Manual of Mental Disorders, Fifth Edition* (DSM-5) [30]; the diagnosis was made by two deputy chief physicians or above in the department of child and adolescent psychiatry. (2) Aged 6–12 years old. (3) Wechsler intelligence measurement ≥ 80. (4) Right-handed. (5) The child and their parent/guardian agreed to participate in this study. The exclusion criteria for the ASD and typically developing (TD) groups were as follows: (1) attention deficit hyperactivity disorder, intellectual development disorder, and other mental diseases; (2) a clear history of neurological diseases, traumatic brain injury, or serious physical diseases; (3) a history of any psychotropic drugs in the past 3 months; (4) metal implants, including irremovable dentures; (5) could not cooperate with the inspection for other reasons. The general information of the subjects is shown in Table 1.

This study was approved by the Ethics Committee of the Brain Hospital affiliated with Nanjing Medical University (No. 2022-KY022-01). The study (including possible risks and benefits of the research) was explained to all subjects and their guardians, after which their signed written informed consent was obtained.

### 2.2. Clinical Psychological Assessment

Professionally trained psychologists or psychiatrists in the Child Psychology Research Center of Nanjing Brain Hospital performed the clinical physiological assessment. A self-developed general information questionnaire was used to collect general data of the subjects, including the subjects’ demographic data, growth experience, disease-related clinical data, history, genetic history, etc. Additionally, the intelligence of the subjects was evaluated using the fourth edition of the Wechsler Scale of Intelligence for Children (WISC-IV) [31]. Moreover, the social interaction and communication abilities of the subjects were assessed using the Autism Diagnostic Observation Schedule (ADOS) [32], the Social Responsiveness Scale (SRS) [33], and the Social Communication Questionnaire (SCQ) [34].

### 2.3. MRI Acquisition

All magnetic resonance data were obtained using a 3-tesla superconducting MRI system (Siemens, Germany) with an 8-channel head coil. During the examination, the subjects were laid on their backs, with cotton balls in both ears and foam pads on their heads. They were asked to remain completely motionless and awake with their eyes closed. All subjects first underwent a routine cross-sectional T2W1 scan for anatomical localization and exclusion of significant intracranial lesions, followed by sagittal three-dimensional SPGR sequencing (3D-SPGR) and rs-fMRI. 

The parameters of the routine MRI sequences were as follows: repetition time (TR) = 2530 ms; echo time (TE) = 3.34 ms; flip angle = 7°; matrix = 256 × 192; field of view (FOV) = 256 × 256 mm; slice thickness = 1.33; and total time = 8 min and 7 s.

The parameters of the rs-fMRI were as follows: TR = 2000 ms, TE = 30 ms, FOV = 256 × 256 mm, matrix: 64 × 64, flip angle = 90°, slice thickness = 4 mm, gap = 0 mm, and total time = 8 min 6 s, with a total of 30 layers and 140 time points acquired.

### 2.4. Data Preprocessing

Preprocessing of fMRI data was performed using Conn Toolbox 20B. The Functional Connectivity Toolkit (https://web.conn-toolkit.org/home (accessed on 15 January 2022)) of a statistical parameter software platform (Statistical Parametric Mapping 12, SPM12: http:∥www.fil.ucl.ac.uk/spm/ (accessed on 17 February 2022)) was used for data preprocessing based on the MATLAB platform. Standard procedures were adopted as follows: (1) Because it takes a certain time for the machine and the subject to enter the stable state, the first 5 time points at the beginning of the scan were removed, while the remaining 135 time points were reserved for subsequent analysis. (2) Slice timing and head movement correction: subjects whose head translation > 2 mm or rotation > 2° were excluded. (3) Standardization: all functional images were registered to the T1 anatomical image and transferred to the space of Montreal Neurological Institute (MNI); the resampling voxel size was 3 × 3 × 3 mm^3^. (4) Bandpass filtering: all images were bandpass filtered in the range of 0.01–0.08 Hz to reduce low-frequency drift and high-frequency physiological noise. (5) The T1 image was divided into gray matter, white matter, and cerebrospinal fluid, and the influence of the head movement was removed using the Friston24 parameter model, while the white matter and cerebrospinal fluid signals were removed by regression. (6) Data smoothing: in order to avoid noise signals, we adopted the Gaussian kernel function to perform smoothing based on 6 mm full width at half-maximum (FWHM).

### 2.5. Social-Related ROIs

Based on the results of previous studies, human social functions are correlated with specific brain regions, including the bilateral posterior inferior temporal sulcus, bilateral prefrontal cortex, bilateral anterior cingulum cortex, bilateral FFG, bilateral THA, bilateral AMYG, bilateral HIP, bilateral superior marginal gyrus, bilateral superior temporal gyrus caudal, bilateral occipital gyrus dorsal mouth, etc. Therefore, the above social-related brain regions were selected as the regions of interest (ROIs) for subsequent analysis. The ROI template was generated using the WFU_PickAtlas tool (https://www.nitrc.org/projects/wfu_pickatlas/ (accessed on 17 February 2022.)).

### 2.6. Functional Connectivity Maps

Seed-based correlation analysis was conducted to analyze abnormal connectivity patterns related to social functions that were significantly different between the two groups. The functional connectivity (FC) map between the seed ROI and the whole brain was calculated in a voxel-by-voxel manner. Specifically, its calculation process was divided into the following three steps: (i) the regionally averaged time-series signal of the seed ROI was obtained as a reference; (ii) the Pearson’s correlation coefficient (PCC) between the time series of each voxel and the reference time series was calculated; (iii) the obtained PCCs were then Fisher r-to-z transformed.

### 2.7. Construction of the Brain Functional Network

**Figure 1:**
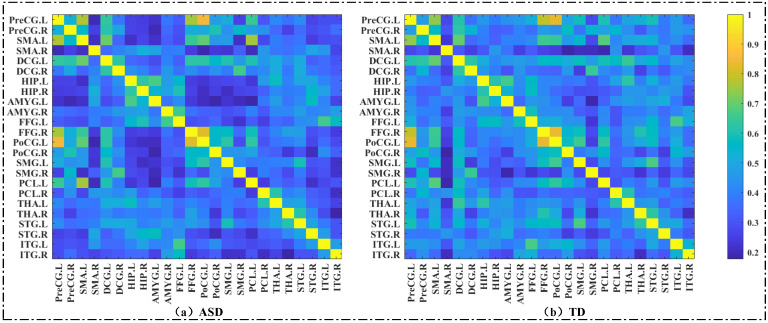
The averaged brain networks of the ASD and TD groups. (**a**) The averaged brain networks of the ASD group. (**b**) The averaged brain networks of the TD group.

**Figure 2:**
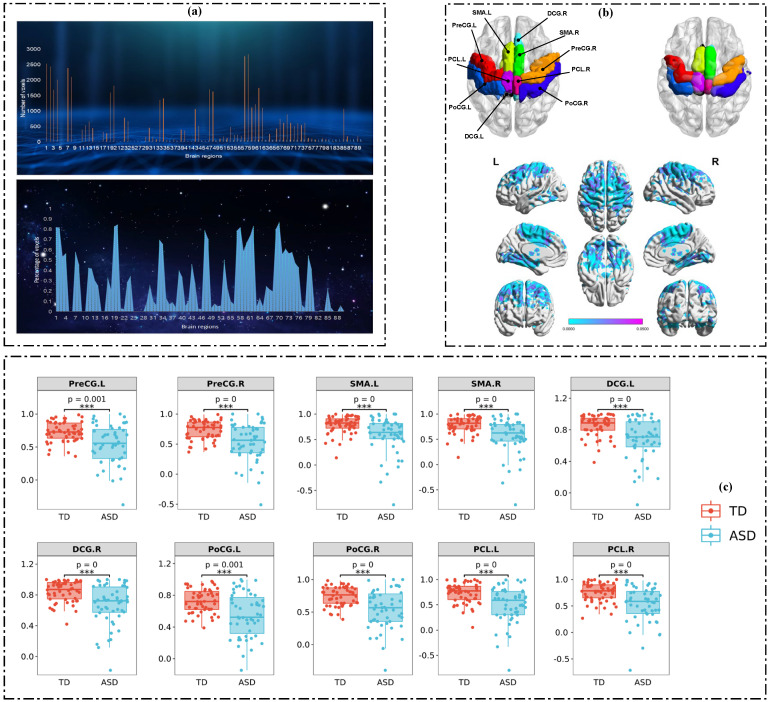
Statistical results of group differences analysis between ASD and TD: (**a**) The statistical results of those voxels with significant group differences. (**b**) The brain mapping results of those voxels with significant group differences. (**c**) The boxplot visualization results of the 10 brain regions with the largest differences.

In addition to the seed-based analysis, network-based analysis was also performed to evaluate the strength of connectivity between brain regions. We regenerated a new ROI template, which contained all of the brain regions originally defined in Section 2.5, and added 10 new brain regions, as shown in Figure 2c. As our voxel-wise analysis results showed that many voxels in these 10 brain regions were associated with functional abnormalities in ASD patients, 10 brain regions were added to the template for further analysis. The new ROI template was reversely mapped to the AAL standard template, and the average time series of these brain regions were extracted. Then, a correlation matrix was obtained by calculating the Pearson’s correlation coefficient between each pair of brain regions, i.e., the brain functional network [35]. The average brain functional networks of all subjects belonging to the ASD group and the TD group are shown in Figure 1.

A one-sample *t*-test was used to detect those edges where the functional connectivity value was significant for the ASD and TD groups. A statistical significance threshold of *p* < 0.05 was assumed, and edges above the threshold formed a connected mask. Comparisons of functional connectivity between the two groups were made within this mask. Network-based statistics (NBS) was used to test for differences between functional connectivity groups.

### 2.8. Brain Network Topology Properties

Two graph metrics—the clustering coefficient and betweenness centrality—were calculated to compare the presence of intergroup differences. The reasons for choosing these two indicators were as follows: (i) the clustering coefficient describes how closely each node is connected to its neighbors, and it is a very efficient metric for measuring the organizational relationships of brain networks; (ii) betweenness centrality is an effective measure for evaluating the roles of nodes in a network. The two complement one another and measure brain networks from different perspectives. Therefore, we selected these indicators to analyze differences in the topological properties of brain networks between different groups of subjects. The detailed definitions of the two graph metrics are as follows:

The clustering coefficient of each node is defined as follows:

$$C_{i}=\frac{2E_{i}}{d_{i}\left(d_{i}-1\right)}$$

where *N* represents the number of nodes in the brain network, *d_i_* denotes the degree of node *i*, *E_i_* is the number of edges connected to node *I,* and *C_i_* is the clustering coefficient of node *i*.

The betweenness centrality of each node is calculated as follows:

$$b_{i}=\frac{1}{\left(N-1\right)\left(N-2\right)}\underset{\begin{array}{c} h,i\in N\\ h\neq j,h\neq i,j\neq i \end{array}}{\sum }\frac{r_{hj}^{\left(i\right)}}{r_{hj}}$$

where *N* is the number of nodes in the network, *r_hj_* is the number of shortest paths between nodes *h* and *j*, and $r_{hj}^{\left(i\right)}$ is the number of paths through node *i* among all shortest paths between nodes *h* and *j*.

### 2.9. Statistical Analysis

#### 2.9.1. Demographic and Clinical Characteristics

The demographic and clinical characteristics were assessed between the two groups to prevent some factors from affecting the objectivity of the results. Age and IQ were compared by χ^2^. Gender was compared using an independent-samples *t*-test.

#### 2.9.2. Voxel-Wise Analysis

Based on the obtained FC maps, we mainly performed two types of analysis: (1) determining which voxels showed significant intergroup differences between the two groups; (2) exploring the correlations between clinical characteristics and the functional connectivity strength.

For the first type of analysis, an independent-samples *t*-test was performed to analyze whether there was a significant difference in the strength of functional connectivity between the two groups, and a *p-*value < 0.05 was considered a significant difference. For the second type of analysis, various clinical characteristics were adopted to explore the correlation of their values with functional connectivity strength. The age, gender, IQ, and mean framewise displacement were taken as covariates in the correlation analysis.

#### 2.9.3. ROI-Wise Analysis

In addition to the voxel-wise analysis, ROI-wise analysis was also performed to evaluate whether there were significant differences in the interaction relationships among social-related brain regions. Based on the new ROI template, we also performed two types of analysis: one to explore whether there were significant differences in the strength of functional connectivity between ROIs in different groups, and the other to determine whether there were significant differences in the graph metrics of brain networks in different groups.

## 3. Results

### 3.1. Demographic and Clinical Characteristics

The results of the demographic and clinical characteristics are shown in Table 1. There were no significant differences in age and gender between the TD and ASD groups (all *p* > 0.05). However, the IQ of the ASD group was significantly lower than that of the TD group (*p* < 0.05). Previous studies have shown that the strength of some functional connectivities and the global efficiency of default networks are significantly associated with intelligence [36,37]. Hence, IQ was used as a covariate in the subsequent test.

### 3.2. Results of Voxel-Wise Analysis

#### 3.2.1. Intergroup Analysis of FC Maps

Figure 2 shows the results of group differences in functional connectivity strength between the two groups. For the analysis results, we performed statistics according to the brain regions of the AAL atlas and showed them in three parts. In Figure 2a, the upper panel reveals the number of voxels with significant differences per brain region. Since the total number of voxels in each brain region may not be consistent, we statistically analyzed the proportion of significantly different voxels in each brain region, i.e., the number of significantly different voxels divided by the total number of voxels in the brain region. The results are shown in the lower panel in Figure 2a. The brain regions with a large number of voxel differences tended to have a relatively high proportion of abnormal voxels, and the overall trend between the two was essentially the same.

Figure 2b shows two types of results: The upper panel shows the spatial distribution of the 10 brain regions with the largest differences between the two groups, derived from the statistical results shown in Figure 2a. The lower panel displays the surface visualization results for regions with *p* < 0.05; the greater the difference, the higher the brightness of those regions. The greatest difference between the two groups was seen at the junction of the temporal lobe and the parietal lobe, indicating that the abnormal function of this region might be the cause of the abnormal behavior of ASD.

The boxplot visualization results of the average connectivity strength across all voxels for the 10 brain regions with the largest differences are shown in Figure 2c. The average functional connectivity strength of the TD group in the 10 brain regions was higher than that of the ASD group (*p* < 0.01).

#### 3.2.2. Correlation between Clinical Characteristics and FC Maps

**Figure 3:**
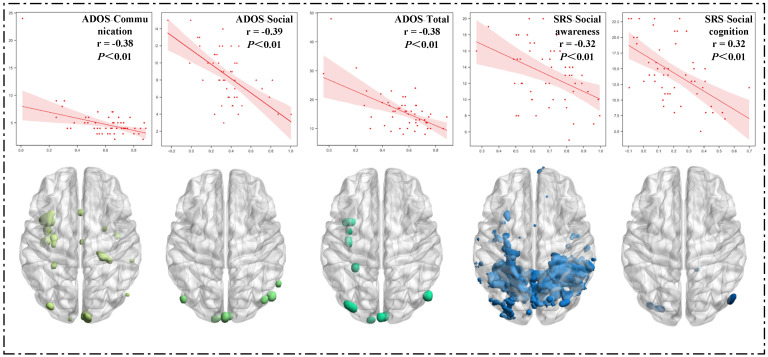
The correlation between clinical characteristics and functional connectivity strength.

Figure 3 presents the significant results of the correlation between clinical characteristics and functional connectivity strength in the ASD group. Specifically, five clinical characteristics were inversely correlated with the FC strength, including ADOS-Communication, ADOS-Social, ADOS-Total, SRS-Social perception, and SRS-Social cognition. In Figure 3, the brain mapping shows that the positions of those voxels are negatively correlated with the corresponding clinical characteristics. Among these five characteristics, the regions with a negative correlation with SRS-Social perception were the largest. From the distribution perspective, all five characteristics seemed to have a larger number of voxels in the left hemisphere than in the right hemisphere. This suggests that the left hemisphere of the ASD group may be more susceptible to disease. Moreover, the FC strength was negatively correlated with the repetitive and stereotyped behavior score on the ADOS scale (r = −0.37, *p* < 0.01).

### 3.3. Results of ROI−Wise Analysis—Graph Metrics of Brain Functional Network

**Figure 4:**
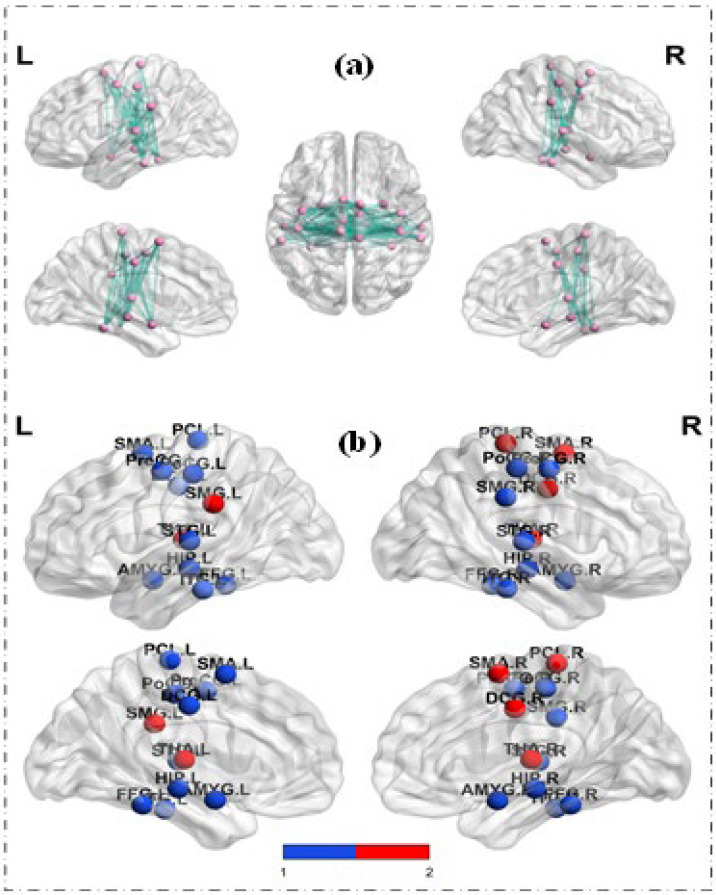
**Statistical results of brain networks:** (**a**) The results of group comparison analysis of the functional connection of brain networks. (**b**) The results of group comparison analysis of the graph metrics.

We constructed a social brain network for each subject based on the new ROI template. As shown in Figure 1, this was a 24 × 24 dimensional matrix. After constructing the brain network, we performed two aspects of the analysis mentioned above. Figure 4a presents those functional connections that were significantly different between TD and ASD, and most of the functional connections between social-related regions were identified as having group differences. Moreover, the results of the group comparison analysis of the graph metrics are shown in Figure 4b. We found group differences in the clustering coefficients of the 24 brain regions. The red nodes did not show significant group differences for betweenness centrality, while the blue nodes showed group differences in betweenness centrality. Table 2 lists the clustering coefficient and betweenness centrality values of these 24 brain regions. The clustering coefficients of the 24 brain regions in the ASD group were smaller than those in the TD group, while betweenness centrality showed the opposite phenomenon, possibly because the dysfunction of some brain regions caused large fluctuations in their roles in information interaction.

## 4. Discussion

The above experimental results (voxel-wise and ROI-wise analysis) showed significant abnormalities in the social-related brain regions of the ASD group compared with the TD group, suggesting that this abnormality may be the potential pathological factor of the ASD group’s lesions.

First, the FC strength between the social-related brain regions and bilateral PreCG was weakened in children with ASD, which is consistent with the findings of most previous studies. The PreCG, situated in the frontal lobe, directly controls the voluntary movement of the body. The frontal lobe is involved in many cognitive functions, such as social cognition, executive function, language, and speech, which are important in interpreting psychosomatic social signals. Previous studies have shown that early dysplasia of the frontal fiber tracts in children with ASD may lead to impaired brain function and affect cognitive flexibility [36], as well as social and communication behaviors [37]. Abnormal activations of the frontal lobe form the neural basis of cognitive flexibility deficits in children with ASD. It is also believed that deficits in cognitive flexibility symbolize the repetitive and stereotyped behavior in individuals with ASD. Therefore, it can be regarded as a “disinhibition phenomenon” combined with the weakened FC in this study. However, Carper et al. found over-connectivity of both the left and right PCG with the prefrontal, parietal, medial occipital, and cingulate cortices [38], which may be explained by the age differences between the subjects and the continuous development and changes of the nervous system. Other studies also support the premise that frontal lobe damage may be a potential neurological trait of social communication disorder in ASD [39]. Moreover, the bilateral PreCG can be a positive pole for improving social deficits and a negative pole for inhibiting repetitive and stereotyped behavior.

Meanwhile, we found a weakening trend in the FC between the social-related brain areas and the bilateral PoCG in children with ASD. The most thoroughly studied somatosensory cortex area is the PoCG, located in the parietal lobe and the somatosensory center. About 70–90% of children with ASD have paresthesia, which may manifest as high or low reactivity to one or more sensory modes. High reactivity is consistent with somatosensory association cortical response, while low reactivity may involve allocating attention to social stimuli or emotions [40]. Moreover, these sensory sensitivities are related to the levels of self-stimulation, social perception, social communication, and social interaction [41], which may aggravate core behaviors of ASD, including stereotyped behavior and social dysfunction [42]. ASD brains have functional whole-brain abnormalities in widespread regions, including the PoCG [43]. The reduced functional connectivity between the left PoCG and the right superior parietal gyrus (SPG) and right superior occipital gyrus (SOG) may be the basis of impaired higher-order multisensory integration in children with ASD, which is related to the severity of symptoms in social and behavioral deficits [44] and is consistent with the results and inference of this study.

FC values showed a weakening trend between the social-related brain areas and bilateral PCL in children with ASD. The PCL is located in the PreCG region on the medial side of the cerebral hemisphere, which is equivalent to the parts of the PreCG and PoCG that fold into the medial side. The anterior part belongs to the frontal lobe, while the posterior part belongs to the parietal lobe. Previously, it was believed that the PCL dominates the motor organs of the feet and calves and is related to urine and stool function. We inferred that lesions to the PCL result in repetitive and stereotyped behavior based on sensation and movement as the basis of the behavior. However, so far, only a few studies have reported on the correlation between PCL lesions and the core symptoms of children with ASD, while there are no relevant studies on social defects. Li et al. showed that the high accuracy of social perception, recognition, and social interaction is related to the activation of the PCL [45]. The FC between the society-related brain regions and the PCL may mediate the clinical severity of social dysfunction in children with ASD. At the same time, the AMYG and PCL interact closely to regulate complex emotional functions [46], and their weakened functional connectivity may be related to the inappropriate social behaviors of children with ASD. Although a previous study demonstrated that individuals with ASD have an increase in transient connectivity between the hypothalamus/subthalamus and some sensory networks (i.e., the right postcentral gyrus, bi-paracentral lobule, and lingual gyrus), this only occurred in certain special functional states, while the global metastate dynamics of the whole-brain functional network diminished similarly [47]. On the whole, this is consistent with the conclusions of our study.

We also found that in children with ASD, the FC between the social-related brain regions and bilateral SMA showed a weakening trend, which is consistent with Wilson’s findings [48]. The SMA is situated in the medial side of the brain hemisphere and in front of the primary motor cortex, which is also mainly related to motor function. Defects in the SMA affect the delayed response function, causing children with ASD to lose their social ability and to become unable to act and communicate consciously [49]. This may explain why ASD children are often addicted to imitation talking. Previous fMRI studies have shown that abnormal patterns of brain function and network connectivity are the main characteristics of ASD, rather than local functional or structural abnormalities. Compared with TD children, the number of functional, connected neural circuits in the SMA is reduced in children with ASD [50], and activation of the SMA is weakened [51], which may be associated with ASD children’s semantic verb processing being inappropriate, resulting in poor pragmatic skills and language ability, and further affecting the children’s behavior and social communication.

Moreover, we found that the FC between the social-related brain areas and the bilateral DCG in children with ASD showed a weakening trend. The cingulum gyrus is an important part of the limbic system with complete fiber connectivity. Detailed studies on the DCG are very limited. However, extensive clinical and experimental data suggest that significant activation of the cingulum gyrus is closely related to attention—especially intentional attention—during conflicting or increasing task difficulty. Therefore, we hypothesized that the weakened functional connectivity between the social-related brain areas and the bilateral DCG might lead to joint attention disorder in children with ASD, thereby affecting their social functions and leading to unconscious stereotyped behavior. However, the detailed mechanism needs to be further studied.

In this study, the clustering coefficient of social-related brain regions in the ASD group was smaller than that in the TD group, indicating that the connectivity between adjacent brain regions was reduced and the ability of local brain regions to process information was decreased in children with ASD, which was roughly consistent with the results of the functional connectivity analysis between social-related brain regions and the whole brain. On the other hand, as for the betweenness centrality of the brain functional networks of social-related brain regions, ASD children showed an increasing trend, except for the right side of the DCG. This also reflected the small-world properties of ASD. On the one hand, this may be because of the aberrant changes in the betweenness centrality of ASD children’s social-related brain regions, with a decreased degree of differentiation between brain regions, which leads to disorder of their social and behavioral function. On the other hand, the changes in the betweenness centrality of social-related brain regions in children with ASD may be the compensative result of their bodies for social and behavioral deficits to enhance the functional integration and differentiated cooperation ability of social-related brain regions.

We found significant intergroup differences in the intensity of functional connectivity between social-related brain regions and four cerebellar regions. Cerebellar FC studies in patients with ASD have been very sparse. Two studies showed increased or decreased FC between “hubs” in the cerebellum and cerebral cortices in ASD groups [52,53]. While the cerebellum has traditionally been thought to control motor behavior, recent studies have shown that cerebellum circuits are essential in illuminating social interactions. As a regulator of mentalization and mirroring processes, the cerebellum participates in the social regulation activities of the cerebral cortex and plays a complicated role in social cognition, such as intention, belief, desire, and personality traits [54]. Although patients with anterior cerebellar impairment often exhibit motor dysfunction, it is unclear whether and how this impairment affects cognitive and social behavior [55]. A previous study took the right cerebellum of children with ASD as a cathode stimulation target for tDCS, finding a 35% improvement in social withdrawal [56]. In the future, the cerebellum could be utilized as a potential target of NIBS to study its social function further.

In general, the weaker the functional connectivity between the abovementioned brain regions and the social-related brain regions with significant intergroup differences, the more severe the clinical symptoms of social deficit and stereotyped behavior in ASD children. This correlation is particularly significant in the communication field of the ADOS scale, social cognition, and the social communication dimension of the SRS scale. These results suggest that abnormal functional connectivity between the social-related and the other aforementioned brain regions is correlated with higher neurological abnormalities, such as social and communication issues. Meanwhile, damage to social networks directly or indirectly leads to stereotyped behavior, which explains why social dysfunction is always accompanied by stereotyped behavior. The difference between the results of this study and those of previous studies may be due to the age differences and IQ differences between the study subjects. It also reflects the heterogeneity of clinical symptoms of children with ASD.

## 5. Study Limitations

Firstly, this study included children aged 6–12 years—mostly boys. Future studies should include children from other age groups, adults, and women with ASD. Secondly, this study was not integrated with clinical treatment strategies. Finally, since this was an exploratory study with a small sample size, the results of the correlation analysis did not pass multiple corrections. Future studies with expanded sample size, NIBS intervention, and follow-up are needed to obtain more reliable conclusions.

## 6. Conclusions

In the present study, we found that the strength of functional connectivity between social-related brain regions and the bilateral PreCG, PoCG, SMA, PCL, and DCG in ASD children showed a significantly weakened trend compared to healthy subjects. In particular, the functional connectivity strength of some brain regions was negatively correlated with clinical scores, and these brain regions were mainly concentrated in the temporal and parietal lobes. The network-based analysis also yielded similar results, indicating that these social-related regions’ functional integration was abnormal. In addition, the dysfunction of these brain regions in the social network always related to behavioral alterations—especially those in the frontal lobe. Since tDCS can enhance the strength of functional connectivity between brain regions, we plan to take these social-related brain regions as potential therapeutic targets for tDCS to improve social dysfunction in ASD children in the future, further verifying the reliability of our results.

## Figures and Tables

**Table 1:** Clinical and demographic characteristics of all participants.

Subject	ASD Group (n = 51)	TD Group (n = 54)	Statistical	*p*
Age (years)	8.16 ± 1.75	8.51 ± 1.82	*t* = −0.999	0.320 ^a^
Gender (male/female)	45/6	45/9	χ^2^ = 0.515	0.473 ^b^
IQ	101.16 ± 17.28	112.96 ± 16.54	*t* = −3.576	0.001 ^a^
ADOS-Communication	5.02 ± 3.13	-	*-*	-
ADOS-Social	8.43 ± 3.08	-	*-*	-
ADOS-Combined social–communication	13.45 ± 5.32	-	*-*	-
ADOS-Total	16.12 ± 6.80	-	*-*	-
SRS-Social awareness	13.02 ± 3.65	-	*-*	-
SRS-Social cognition	14.49 ± 4.87	-	*-*	-
SRS-Social communication	21.84 ± 9.66	-	*-*	-
SRS-Social motivation	13.86 ± 4.83	-	*-*	-
SRS-Autistic mannerisms	13.10 ± 5.21	-	*-*	-
SRS-Total	76.31 ± 17.97	-	*-*	-
SCQ-Total	22.14 ± 6.14	-	*-*	-

All data are presented as the mean ± standard deviation. ^a^ Independent-samples *t*-test; ^b^ chi-squared test.

**Table 2:** Clustering coefficients and betweenness centrality of 24 social-related brain regions in the ASD and TD groups.

	Attributes Groups	Clustering Coefficient		Betweenness Centrality	
Brain Regions		ASD	TD	ASD	TD
PreCG.L		0.41 ± 0.25	0.61 ± 0.15	96.96 ± 155.99	9.35 ± 58.83
PreCG.R		0.40 ± 0.25	0.58 ± 0.16	110.10 ± 171.46	13.67 ± 67.71
SMA.L		0.40 ± 0.25	0.59 ± 0.15	102.78 ± 172.31	19.41 ± 87.20
SMA.R		0.36 ± 0.20	0.48 ± 0.17	103.06 ± 148.17	78.80 ± 139.31
DCG.L		0.45 ± 0.23	0.62 ± 0.14	52.04 ± 117.89	0.52 ± 2.99
DCG.R		0.42 ± 0.21	0.55 ± 0.17	45.88 ± 116.65	50.22 ± 116.59
HIP.L		0.39 ± 0.22	0.58 ± 0.15	103.12 ± 160.20	4.31 ± 18.94
HIP.R		0.38 ± 0.23	0.56 ± 0.15	110.57 ± 164.67	16.19 ± 50.77
AMYG.L		0.35 ± 0.22	0.56 ± 0.16	139.04 ± 166.83	23.78 ± 77.79
AMYG.R		0.40 ± 0.23	0.56 ± 0.16	92.78 ± 150.58	29.59 ± 93.00
FFG.L		0.39 ± 0.25	0.58 ± 0.15	127.08 ± 172.05	20.07 ± 75.18
FFG.R		0.42 ± 0.24	0.60 ± 0.15	95.29 ± 142.22	9.81 ± 41.27
PoCG.L		0.41 ± 0.25	0.60 ± 0.15	107.31 ± 146.61	16.37 ± 56.40
PoCG.R		0.38 ± 0.25	0.56 ± 0.15	137.76 ± 164.96	21.11 ± 80.07
SMG.L		0.39 ± 0.24	0.56 ± 0.16	98.55 ± 147.10	64.31 ± 134.05
SMG.R		0.35 ± 0.23	0.50 ± 0.16	148.02 ± 181.61	74.17 ± 125.39
PCL.L		0.38 ± 0.24	0.57 ± 0.15	123.67 ± 161.13	24.81 ± 70.56
PCL.R		0.37 ± 0.21	0.55 ± 0.18	77.31 ± 134.41	56.87 ± 141.81
THA.L		0.41 ± 0.22	0.57 ± 0.17	67.53 ± 146.93	45.46 ± 115.43
THA.R		0.38 ± 0.22	0.56 ± 0.17	94.27 ± 155.80	53.09 ± 130.08
STG.L		0.43 ± 0.22	0.60 ± 0.16	68.00 ± 129.87	13.46 ± 62.22
STG.R		0.37 ± 0.22	0.54 ± 0.17	97.63 ± 137.11	33.33 ± 72.49
ITG.L		0.38 ± 0.25	0.58 ± 0.15	113.90 ± 154.18	4.93 ± 27.41
ITG.R		0.33 ± 0.25	0.53 ± 0.17	151.20 ± 171.27	52.33 ± 116.76

The abbreviations of the brain area names are consistent with the AAL template. Data are presented as the mean ± standard deviation.

## Data Availability

Raw data is unavailable due to privacy. Readers can request the data without patients’ personal information from the corresponding author.
